# Multi-omics approach to study the dual effects of novel proteins on the intestinal health of juvenile largemouth bass (*Micropterus salmoides*) under an alternate feeding strategy

**DOI:** 10.3389/fimmu.2023.1110696

**Published:** 2023-03-01

**Authors:** Lukuan Li, Yu Wang, Yanqing Huang, Chunfang Wang

**Affiliations:** ^1^ Engineering Research Center of Green Development for Conventional Aquatic Biological Industry in the Yangtze River Economic Belt, Ministry of Education, Hubei Provincial Engineering Laboratory for Pond Aquaculture, College of Fisheries, Huazhong Agricultural University, Wuhan, China; ^2^ Key Laboratory of Inland Saline-Alkaline Aquaculture, Ministry of Agriculture and Rural Affairs, East China Sea Fisheries Research Institute, Chinese Academy of Fishery Sciences, Shanghai, China

**Keywords:** intestinal health, alternate feeding strategy, intestinal barrier, novel protein diets, largemouth bass, SCFAs (short chain fatty acids)

## Abstract

**Introduction:**

In an effort to minimize the usage of fishmeal in aquaculture, novel protein diets, including Tenebrio molitor, cottonseed protein concentrate, *Clostridium autoethanogenum*, and *Chlorella vulgaris* were evaluated for their potential to replace fishmeal. Nevertheless, comprehensive examinations on the gut health of aquatic animals under an alternate feeding strategy when fed novel protein diets are vacant.

**Methods:**

Five isonitrogenous and isolipidic diets containing various proteins were manufactured, with a diet consisting of whole fishmeal serving as the control and diets containing novel proteins serving as the experimental diets. Largemouth bass (Micropterus salmoides) with an initial body weight of 4.73 ± 0.04g employed as an experimental animal and given these five diets for the first 29 days followed by a fishmeal diet for the next 29 days.

**Results:**

The results of this study demonstrated that the growth performance of novel protein diets in the second stage was better than in the first stage, even though only the *C. vulgaris* diet increased antioxidant capacity and the cottonseed protein concentrate diet decreased it. Concerning the intestinal barriers, the *C. autoethanogenum* diet lowered intestinal permeability and plasma IL-1β/TNF-α. In addition, the contents of intestinal immunological factors, namely LYS and sIgA-like, were greater in *C. vulgaris* than in fishmeal. From the data analysis of microbiome and metabolome, the levels of short chain fatty acids (SCFAs), anaerobic bacteria, *Lactococcus*, and Firmicutes were significantly higher in the *C. autoethanogenum* diet than in the whole fishmeal diet, while the abundance of *Pseudomonas*, aerobic bacteria, *Streptococcus*, and Proteobacteria was lowest. However, no extremely large differences in microbiota or short chain fatty acids were observed between the other novel protein diets and the whole fishmeal diet. In addition, the microbiota were strongly connected with intestinal SCFAs, lipase activity, and tight junctions, as shown by the Mantel test and Pearson’s correlation.

**Discussion:**

Taken together, according to Z-score, the ranking of advantageous functions among these protein diets was *C. autoethanogenum* diet > *C. vulgaris* diet > whole fishmeal diet > cottonseed protein concentrate > *T. molitor* diet. This study provides comprehensive data illustrating a mixed blessing effect of novel protein diets on the gut health of juvenile largemouth bass under an alternate feeding strategy.

## 1 Introduction

Aquaculture provides humans with enormous amounts of high-quality proteins. Both oceanic and freshwater aquaculture need vast quantities of fishmeal, a high-quality protein for aquaculture (1). However, fishmeal is unsustainable, from 2020 FAO report, the yearly output of fishmeal in the next decade may rise by no more than 1% compared to 2018, while the price of fishmeal will climb by 30%, putting a significant strain on diets prices and worldwide fishmeal stock capacity (2). Consequently, it is crucial to explore high-quality fishmeal substitutes in aquatic practices.

*Chlorella vulgaris* diet, *Clostridium autoethanogenum* protein diet, cottonseed protein concentrate diet, and *Tenebrio molitor* diet are developed to ease the shortage of fishmeal in feed industry. In recent years, researchers have concentrated on these novel proteins owing to their abundant fat/fatty acids, low price, high content of protein, mineral, balanced amino acid profiles, and vitamin content (3–6). Earlier studies have examined the impacts of these proteins on *Cyprinus carpio* var. *specularis*, *Litopenaeus vannamei*, and *Micropterus salmoides* (4, 7–9). Nonetheless, these studied mainly focus on partial substitution strategy of fishmeal, and explore their functions on muscle quality, growth performance and intestinal health. Few studies have been carried out to investigate in depth the impacts of novel proteins on fish growth performance and intestinal immunity from the perspective of alternate feeding strategy while comparing the relative substitution potential of each novel protein diet.

Alternate feeding strategy is an effective method to conserve high-value or resource-limited feed ingredients. Some studies have extensively conducted for fish oil based diet substitution with terrestrially derived oil or plant-sourced oil diets without compromising fish fatty acid composition (10), fish growth performance (11), or their physio-biochemical performance (12, 13) in *Scophthalmus maximus*, *Gadus morhua*, *Dicentrarchus labrax*, and *Acanthopagrus schlegelii*. These studies showed that using an alternate feeding strategy could reduce the use of fish oil. However, few studies have focused on replacing fishmeal with novel proteins under an alternate feeding strategy. The intestine is the primary location for the absorption and digestion of fish nutrients, and its health is favorable to the correct execution of fish physiological processes (14). It is commonly accepted that the intestinal barriers include of immunological, microbial, physical, and chemical barriers (15), which play key roles in fish growth, nutritional status, immunology, and resistance to illness.

**Figure 1:**
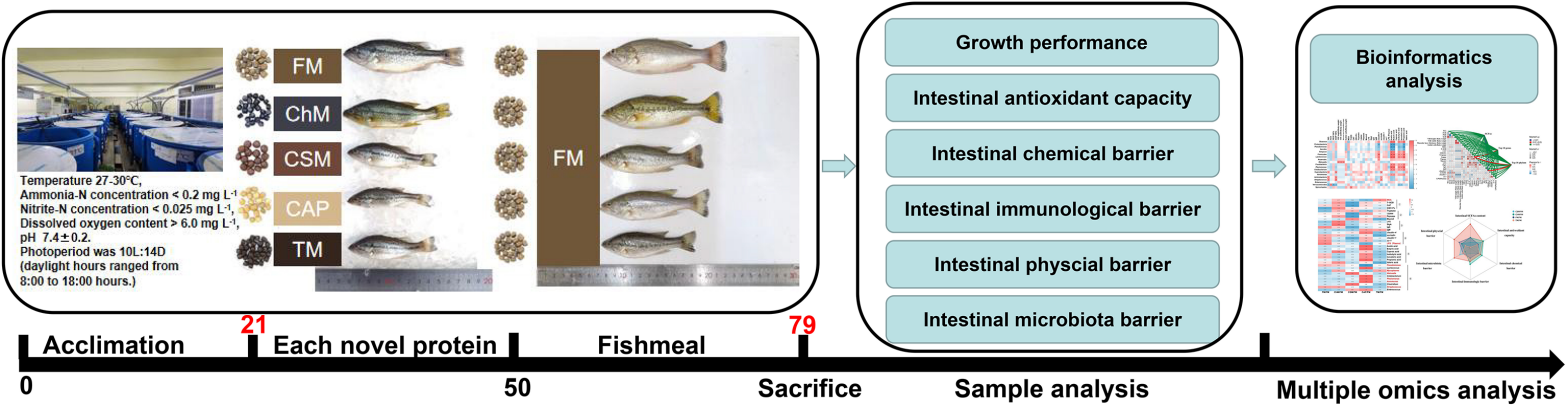
The work flow of this study. Whole fishmeal diet, *C. vulgaris* diet, cottonseed protein concentrate, *C. autoethanogenum* diet, and *T. molitor* diet, referred as FM/FM, ChM/FM, CSM/FM, CAP/FM, and TM/FM, respectively.

Largemouth bass (*M. salmoides*), which is one of the most cultivated species in China, was selected as a research model owing to its high fishmeal consumption in commercial diets, which ranges between 35 and 50 percent (16). In order to explore novel protein diets that might potentially replace fishmeal in fish diets, this research aims to evaluate the effects of novel protein diets on the gut health and growth performance of juvenile largemouth bass under an alternate feeding approach. Compared to previously published research on these proteins, the present study displayed a comprehensive view of the interaction between novel proteins and gut health and growth performance, with a focus on the connection between gut microbiota, their metabolites, and physiochemistry biomarkers under an alternate feeding strategy, as illustrated in **Figure 1**.

## 2 Materials and methods

### 2.1 Experimental design

The methodologies employed in this research properly adhered to the management Rule of Laboratory Animals (Chinese order no. 676 of the State Council, revised 1 March, 2017). The largemouth bass were provided by Hubei Zhenghao Fish Fry farm. Cottonseed protein concentrate, Fishmeal, *C. autoethanogenum*, *T. molitor* and *C. vulgaris* were formulated to five isonitrogenous and isolipidic expanded pellet diets. The approximate composition was shown in **Supplementary Table 1**. Each feed was molded into 2 mm floating pellets and air-dried for two days at room temperature. All experimental diets were kept at a temperature of -20°C. Anterior to the start of the growth trial, the five diets were pooled and supplied to juvenile largemouth bass for 21 days to prevent the unpalatability and to acclimatize them to the experimental surroundings. Five groups of largemouth bass (starting body weight: 4.73 ± 0.04g) with three replicates per treatment were allocated to 15 blue cylindrical acrylic tanks (Water volume: 400 L) at random, containing 35 fish per tank in a recirculation system. The experiment consisted of two stages, in which fish were initially given novel proteins for 29 days, then fishmeal for another 29 days, and all were fed twice daily at 9:00-9:30 am and 5:00-5:30 pm until a level of apparent satiety. The bulk body weight of fish was recorded every week. Throughout the duration of the growth trial, about 1/7 of the water was replenished every day to remove faeces buildup. During the growth trial, the water quality was maintained as follows: 27-30°C water temperature, ammonia-N<0.2 mg L^-1^, nitrite-N<0.025 mg L^-1^, and dissolved oxygen>5.0 mg L^-1^, pH at approximately 7.5, and 10L:14D photoperiod with illumination from 8:00 am to 6:00 pm.

### 2.2 Sample collection

In order to examine the gut microbiota and short chain fatty acids (SCFAs), seven fish from each treatment were randomly taken after a 6-hour fast on day 58 and anesthesia with 75 mg L^-1^ MS-222 (Aladdin, Shanghai, China). The method for sacrificing fish was previously described (17). The entire contents of the intestine were collected in sterile centrifuge tubes, which were immediately frozen in liquid nitrogen and stored at -80°C. Three samples from each treatment, with similar bacterial structures and compositions, were analyzed for their SCFAs contents. On day 58, additional fish were starved for 24 hours, anesthetized with the same amount of MS-222, slaughtered, and dissected as described above. The middle intestine of nine fish from each treatment was rapidly frozen in liquid nitrogen and stored at -80°C for investigation of intestinal biochemical indexes and associated gene expression. For morphological study, the distal intestines of nine fish from each treatment group were preserved in 4% paraformaldehyde. For intestinal permeability investigations, serum was taken from nine fish per treatment, allowed to rest at 4°C for overnight, and the supernatant was collected. The initial body weight (IBW), final body weight (FBW), average daily gain (ADC), Feed conversion ratio (FCR), protein efficiency ratio (PER), specific growth rate (SGR), and survival rate (SR) were calculated using the following formulae:


ADC=(Final fish weight−Initial fish weight)/Number of days

FCR=(Feed intake/Weight gain)

PER(%)=100×(Fresh body weight gain)/(Dry feed intake×Protein of feed)

SGR (%)={[ln (Final body weight)–ln (Initial body weight)]/Number of days}

SR(%)=100×(Final fish number/Initial fish number)

### 2.3 Examining the intestinal morphology and hematological parameters

The intestinal tissue slice slides were made by Service bio-Company (Wuhan, China). Briefly, the middle gut was washed in sterile saline, fixed for 48 hours in 4% paraformaldehyde, and then rinsed in 70% ethanol. 5μm thick slices of paraffin-embedded, aematoxylin-eosin-stained intestines. Using an Olympus DP72 microscope paired with a Nikon E600 camera and Olympus CellSens Standard software to acquire images. At 10 sites on a single slide, the height/width of each villi, the muscle layer thickness and the number of goblet cells, were assessed. In addition, in order to eliminate the effects from body length, the quantification results from morphology were calibrated. Using commercial Elisa kits, the serum concentrations of lipopolysaccharide (LPS), lysozyme (LYS), Interleukin 1 beta (IL-1β), and tumor necrosis factor (TNF-α) were measured (Enzyme-linked Biotechnology, Shanghai, China). The required operation was conducted in accordance with the instructions.

### 2.4 Evaluation of the intestinal biochemical parameters

Approximately 100 mg of intestinal tissue was mixed with cold saline to generate a 10% homogenate (Tissue: Saline=1:9). After centrifugation (4500 rpm/min) for 15 minutes, the supernatant was collected and analyzed. Employing commercial test kits (Nanjing jiancheng Bioengineering Institute, Nanjing, China), the activities of superoxide dismutase (SOD), catalase (CAT), and malondialdehyde (MDA) were determined. Utilizing commercial Elisa kits, the level of glutathione peroxidase (GSH-Px) and mucin 2 (MUC2), lysozyme (LYS), immunoglobulin M (IgM), immunoglobulin G-like (IgG-like), and secretory immunoglobulin-like (sIgA-like) were determined (Enzyme-linked Biotechnology, Shanghai, China). The required operation was conducted in conformity with the instructions.

### 2.5 Real-time fluorescent quantitative PCR

RT-qPCR was conducted as previously described (17). Briefly, total RNA of middle intestine was extracted, and RNA templates were used to transcribe cDNAs. The cDNAs were amplified by PCR using the PrimeScript RT reagent Kit (Takara, Japan). With the primers provided in **Supplementary Table 2**, the RT-qPCR was conducted on a CFX96™ Real-Time System (bio-RAD, USA). *β-actin* was chosen as a housekeeping gene due to its stable expression. The primers, i.e., *occludin*, *claudin-1*, *zo-1*, *claudin-4*, *sod*, *cat*, *gpx*, *keap-1*, and *nrf-2*, were selected and exhibited amplification efficiencies ranging from 91% to 115%. The results of real-time RT-qPCR were analyzed by the 2^−ΔΔCT^ method.

### 2.6 Microbiome study based on sequencing of the 16S rRNA gene

Following the manufacturer’s instructions, total bacterial DNA was extracted from the intestinal contents using an OMEGA DNA Kit (D5625-01) (Omega Bio-Tek, Norcross, GA, USA). PCR amplification and Illumina MiSeq sequencing (Shanghai, China) were used to study the V3-V4 region of the bacterial 16S rRNA gene. Refer to the additional materials for more information.

### 2.7 Measurement of short chain fatty acids

The detection of intestinal contents short chain fatty acids was accomplished by the previously described technique of gas chromatography-mass spectrometry (GC-MS) (17). See supplementary materials for details.

### 2.8 Data analysis

SPSS 25.0 (IBM, Armonk, NY, USA) were used to analyze the data. For data not normally distributed, the nonparametric Krustal-Wallis test followed by the Dunn’s multiple comparison test was applied. One-way ANOVA was used to evaluate normally distributed data, following with least significant differences for multiple treatments comparisons. *P<0.05* was considered statistical differences. Illustrations were created using R 3.6.1 ggplot2 package as well as GraphPad Prism 8.3.0. In addition, we supported the idea that statistical differences should not serve as the exclusive criteria for assessing data discrepancies (18), and we wish to apply multiple criterion to study data discrepancies among experimental treatments fully. Consequently, z-score value was employed to standardize the data and it was used to assess the complex data and their complicated association owing to the huge amount of differently classified physiological indicators utilized in our study. In addition, Z value is negative once the original value is below the mean and positive when the original value is above the mean. This Z value is independent of the original unit of measurement.

## 3 Results

### 3.1 Growth performance, feed utilization and intestinal histological analysis of juvenile largemouth bass under an alternate feeding strategy

**Figure 2:**
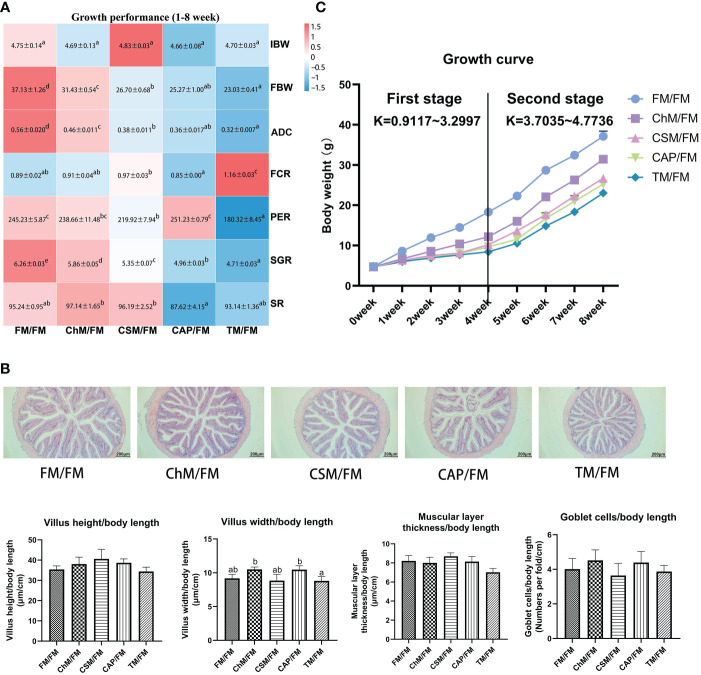
The growth performance and intestinal morphology of juvenile largemouth bass under an alternate feeding strategy. Growth performance heatmap **(A)**;The distal intestines of FM/FM, ChM/FM, CSM/FM, CAP/FM, and TM/FM protein diet under 100 x magnification and the intestinal quantitative data. **(B)**; Wight gain growth curve **(C)**. Data are also expressed as mean (S.E.M). Values on each column/heatmap with different superscripts typified statistical differences (*P<0.05*).

As demonstrated in **Figures 2A, B**, FBW, ADC, SGR, and PER were reduced in diets containing novel proteins, although the SR, intestinal morphology, villus structure, muscular layer thickness, and goblet cells were unaltered. However, significant changes in FCR were not seen in ChM/FM, CSM/FM, and CAP/FM. **Figure 2C** demonstrates that the slopes of K of 0-4 weeks were less than the 4-8 weeks, which showed that growth performance of treatments containing novel protein diets in the second stage outperformed the first stage.

### 3.2 The oxidative stress of juvenile largemouth bass under an alternate feeding strategy

**Figure 3:**
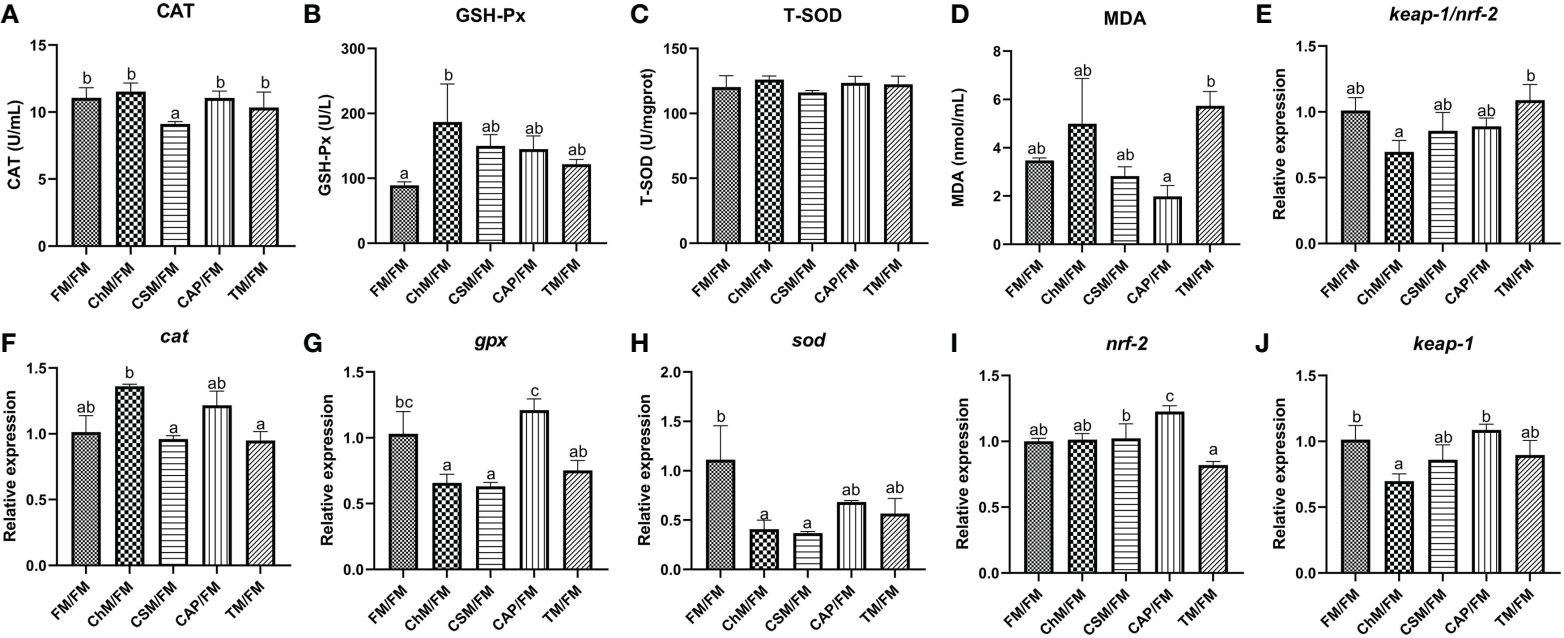
The antioxidant enzymes and genes expression of juvenile largemouth bass intestines under an alternate feeding strategy were shown as follows: CAT **(A)**, GSH-Px **(B)**, T-SOD **(C)**, MDA **(D)**, *keap-1*/*nrf-2* **(E)**, *cat* **(F)**, *gpx* **(G)**, *sod* **(H)**, *nrf-2* **(I)**, and *keap-1* **(J)**. Data are expressed as mean (S.E.M). Values on each column with different superscripts typified statistical differences (*P<0.05*).

The levels of CAT, T-SOD, MDA, GSH-Px, *cat*, *gpx*, *sod*, *nrf-2*, and *keap-1* were evaluated in the intestine (**Figures 3A–J**). With the greatest GSH-Px activity in the ChM/FM group and the lowest CAT activity in the CSM/FM group, there were no dramatically discrepancies in the levels of MDA and T-SOD between diets containing novel proteins and FM/FM. In comparison to other diets, the levels of *gpx* and *sod* were significantly reduced in the ChM/FM and CSM/FM groups. Although the expression patterns of *nrf-2* and *keap-1* were distinct, the ratio of *keap-1/nrf-2* did not alter substantially between diets containing novel proteins and FM/FM. These non-significant variations were also discovered in the level of *cat*.

### 3.3 The intestinal chemical barriers and immunologic barriers of juvenile largemouth bass under alternate feeding strategy

**Figure 4:**
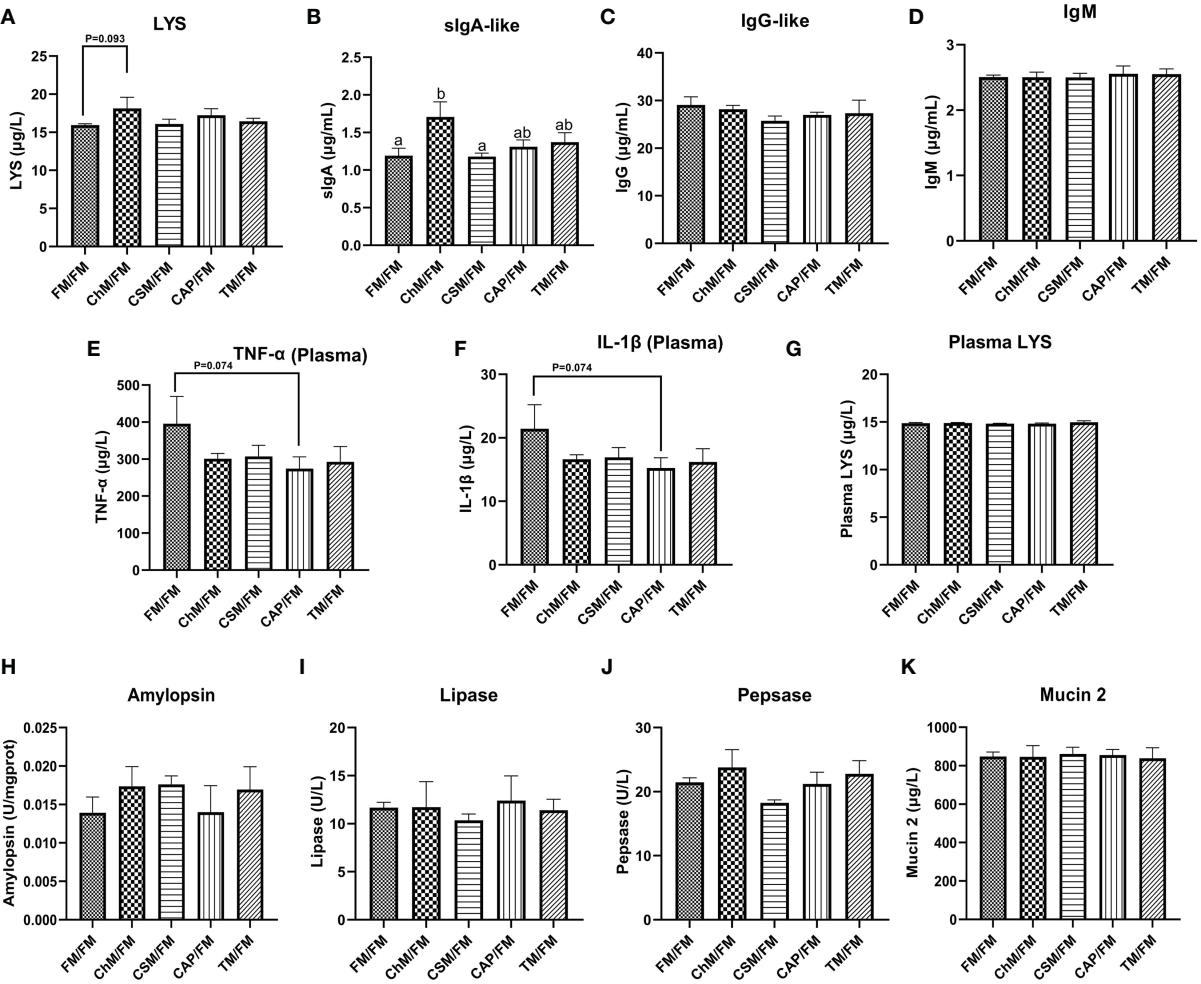
The immunological and chemical barriers of juvenile largemouth bass under an alternate feeding strategy were shown as follows: LYS **(A)**, sIgA-like **(B)**, IgG-like **(C)**, IgM **(D)**, plasma TNF-α **(E)**, plasma IL-1β **(F)**, LYS **(G)**, amylopsin **(H)**, lipase **(I)**, pepsase **(J)**, and mucin 2 **(K)**. Data are expressed as mean (S.E.M). Values on each column with different superscripts typified statistical differences (*P<0.05*).

The contents of LYS, sIgA-like, IgG-like, IgM, TNF-α, IL-1β, amylopsin, lipase, pepsase, and mucin 2 in the gut were examined to further investigate the impacts of alternate feeding of diets containing novel proteins on immunochemistry barriers (**Figures 4A–K**). Except for sIgA-like, no statistically significant alterations were seen in these biochemical indices. However, the plasma concentrations of TNF-α and IL-1β were lower in CAP/FM than in FM/FM (P=0.074). Moreover, the greatest levels of two crucial immunological markers, LYS and sIgA-like, were seen in the ChM/FM group.

### 3.4 The intestinal permeability of juvenile largemouth bass under an alternate feeding strategy

**Figure 5:**
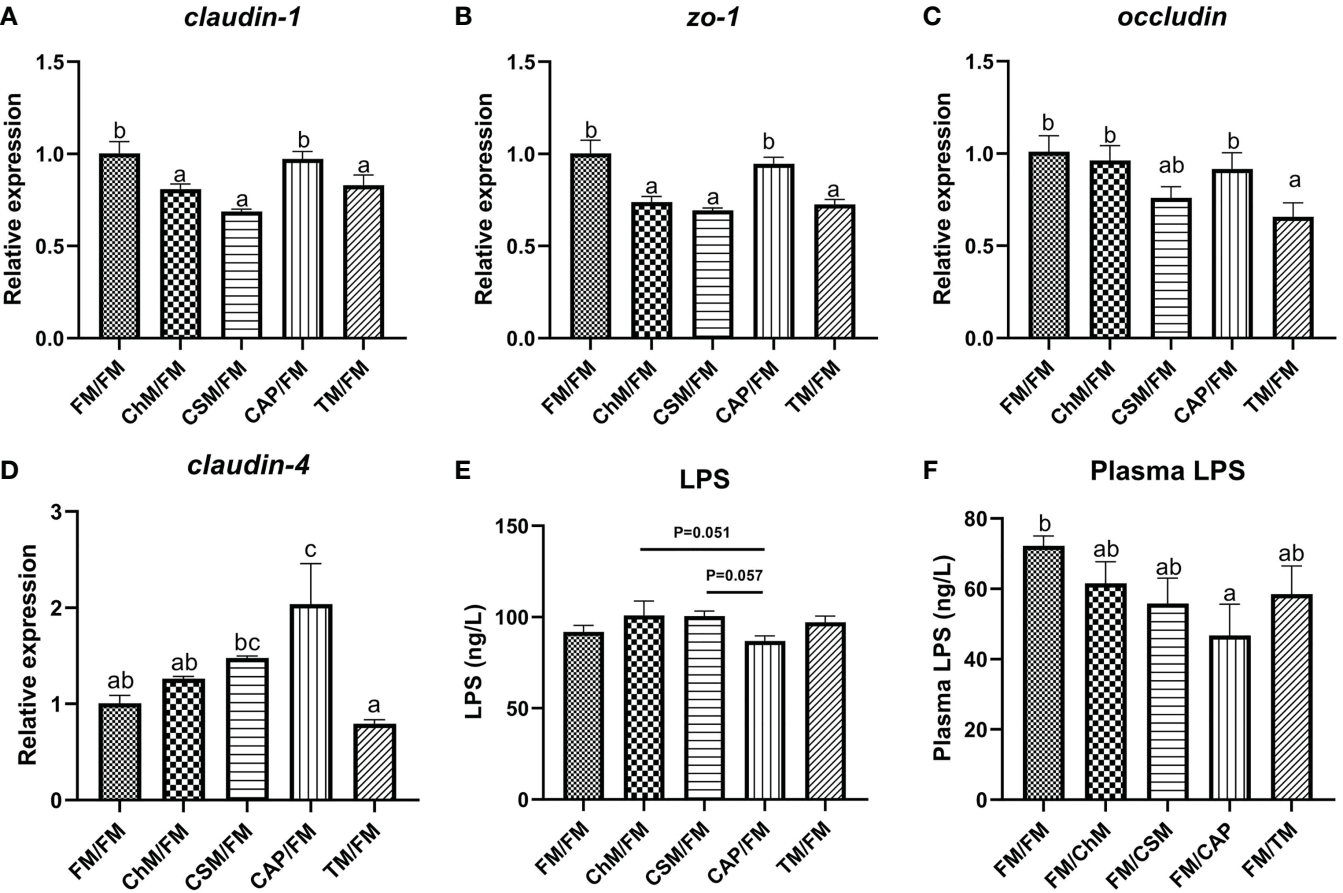
The intestinal physical barrier of juvenile largemouth bass under an alternate feeding strategy were shown as follows: *claudin-1* **(A)**, *zo-1* **(B)**, *occludin* **(C)**, *claudin-4* **(D)**, intestinal LPS **(E)**, and plasma LPS **(F)**. Data are expressed as mean (S.E.M). Values on each column with different superscripts typified statistical differences (*P<0.05*).

The intestinal permeability was measured to assess the intestinal physical barrier’s integrity (**Figures 5A–F**). The level of *claudin-1* and *zo-1* did not differ statistically between CAP/FM and FM/FM, however the expression of these genes in other protein diets was considerably reduced in comparison to FM/FM (*P<0.05*). Intriguingly, the level of *claudin-4* was substantially greater in CAP/FM than in FM/FM (*P<0.05*), and the transcription of *occludin* was significantly lower in TM/FM (*P<0.05*). In addition, the plasma LPS concentrations of all diets containing novel proteins were lower than FM/FM, and the intestinal LPS content of CAP/FM was lower than CSM/FM (*P=0.057*) and ChM/FM (*P=0.051*), while there were no significant differences amongst the five protein diets.

### 3.5 Intestinal microbiome analysis of juvenile largemouth bass under an alternate feeding strategy

**Figure 6:**
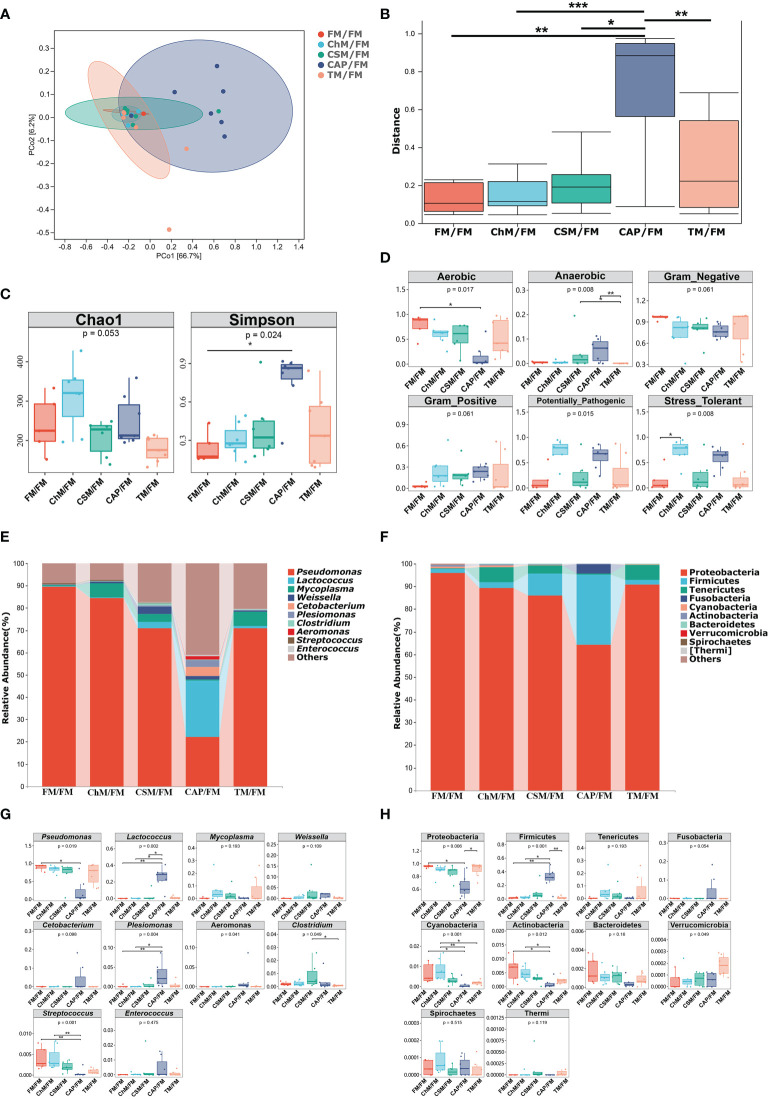
The intestinal microbiome of juvenile largemouth bass intestinal contents under an alternate feeding strategy were shown as follows: β-diversity **(A)**, abundance comparison of β-diversity **(B)**, α-diversity **(C)**, bugbase analysis **(D)**, top 10 microbiota at genus level **(E)**, Top 10 microbiota at phylum level **(F)**, abundance comparison of top ten microbiota at genus level **(G)**, and abundance comparison of top 10 microbiota at phylum level **(H)**.The statistical differences were presented as *P<0.05,**P<0.01, and ***P<0.001.

To better comprehend the impact of new protein diets on the intestinal microbiome of juvenile largemouth bass under an alternative feeding strategy, the microbial diversity, functions, and compositions were investigated. **Figures 6A–C** demonstrate that the β-diversity of CAP/FM is substantially greater than that of other diets (*P<0.05*). Similarly, the α-diversity of CAP/FM exhibited a greater Simpson index than FM/FM (*P<0.05*). The bugbase function and phenotypic predictions (**Figure 6D**) revealed that ChM/FM had more stress tolerance than all other diets. Additionally, the quantity of anaerobic bacteria in CAP/FM exceeded that of the other protein diets, but the abundance of aerobic bacteria was the lowest. **Figures 6E, F** illustrate the intestinal composition at both the phylum and genus levels. Firmicutes, a SCFAs-producing phylum, and Proteobacteria, a phylum of opportunistic pathogens, changed significantly. Accordingly, the quantity of probiotics (*Lactococcus*) and pathogens (*Pseudomonas*) in the five protein diets was highly variable at the genus level. Specifically, **Figures 6G, H** depict the comparison of abundance of microbiota. The CAP/FM diet included the greatest abundance of *Lactococcus* and Firmicutes and the lowest abundance of *Pseudomonas* and Proteobacteria. In addition, CAP/FM showed a greater abundance of *Plesiomonas* than both ChM/FM and FM/FM (*P<0.05*). In contrast, these two protein diets included a greater number of Actinobacteria, *Streptococcus*, and Cyanobacteria than the CAP/FM diet. However, other microbiota did not differ considerably between diets containing novel proteins and FM/FM.

### 3.6 The contents of SCFAs in juvenile largemouth bass under an alternate feeding strategy

**Figure 7:**
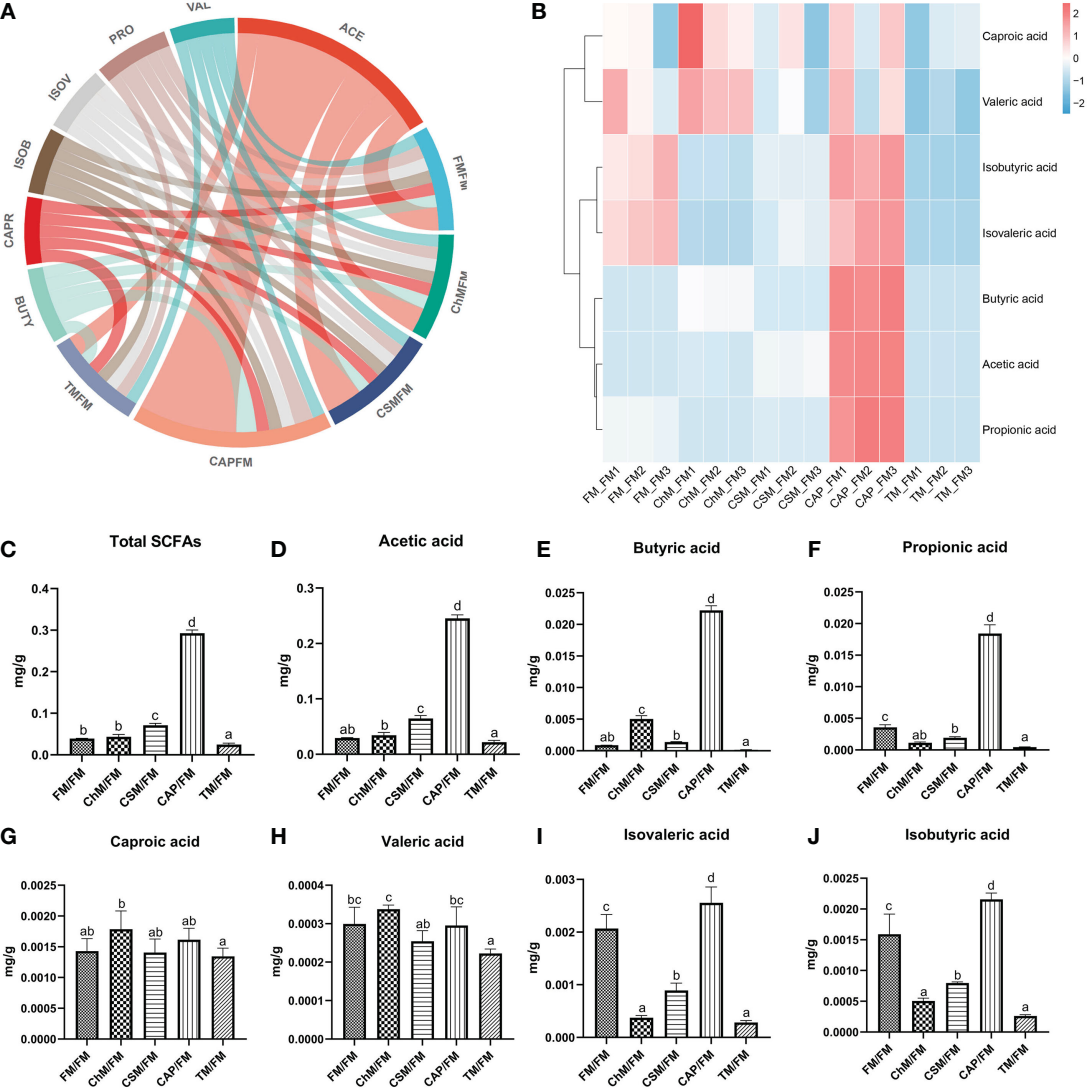
The intestinal metabolome of juvenile largemouth bass intestinal contents under an alternate feeding strategy were shown as follows: Distribution of SCFAs in each diet **(A)**, relative abundance of SCFAs in each diet **(B)**, total SCFAs contents **(C)**, acetic acid content **(D)**, butyric acid content **(E)**, propionic acid content **(F)**, caproic acid content **(G)**, valeric acid content **(H)**, isovaleric acid content **(I)**, and isobutyric acid content **(J)**. Data are expressed as mean (S.E.M). Values on each column with different superscripts typified statistical differences (*P<0.05*).

SCFAs have crucial roles in maintaining intestinal health and preventing occurrence of disease; thus, their presence in the five protein diets were considered. **Figure 7A** is a chord diagram depicting the contents of intestinal SCFAs in each protein diet. Seven SCFAs were discovered, with the greatest acetic acid concentration and the lowest isobutyric acid level. The heatmap in **Figure 7B** reveals that the CAP/FM diet included a greater proportion of SCFAs than other protein diets. In contrast, the concentration of SCFAs in TM/FM was the lowest. The bar graphs clearly (**Figures 7C–J**) demonstrate the considerable variation in SCFAs levels across all protein diets (**Figure 7C**). The lowest levels of total SCFAs, acetic acid, butyric acid, and propionic acid were found in the TM/FM diet, whereas the highest levels were found in the CAP/FM diet. Comparatively, the concentrations of isovaleric acid and isobutyric acid in the CAP/FM diet were considerably greater than in the FM/FM diet (*P<0.05*), although these two acids were significantly lower in other diets containing novel proteins (*P<0.05*). Additionally, no considerable differences were identified between diets containing novel proteins and FM/FM for caproic acid and valeric acid.

### 3.7 The integrity analysis of juvenile largemouth bass under an alternate feeding strategy

**Figure 8:**
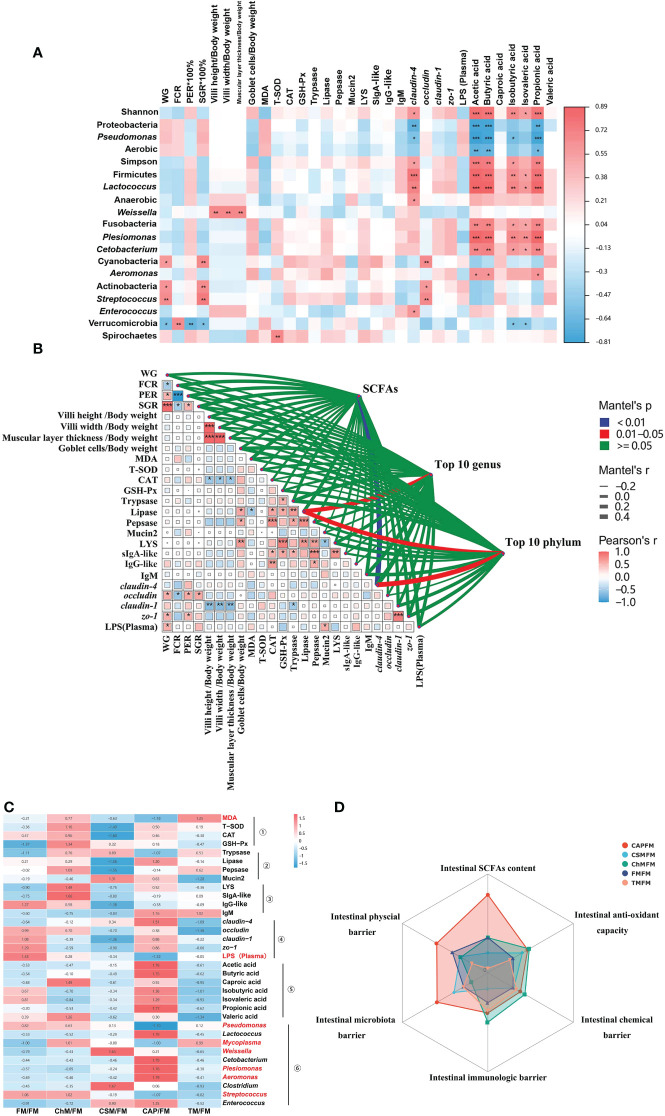
Combined analysis and scoring of all data obtained from various protein treatments: **(A)** Heat map of Pearson’s correlation analysis (|r|>0.6, P 0.05) of bacterial phenotype, abundance at the genus/phylum level, SCFAs, and physiological biomarkers. On the heat map, the red square indicates a positive association, while the blue square indicates a negative correlation. **(B)** The Mantel test was performed on the core microbiota, intestinal SCFAs, and physiological indicators. The size of the square in the matrix symbolizes the coefficient value, with red squares representing positive correlations and blue squares negative correlations. The lines outside of the matrix reveal the relationship between the core microbiota as well as intestinal SCFAs and physiological markers, with thicker lines indicating stronger associations. **(C)** Heat map of the z-score analysis was clustered as the intestinal antioxidant①, intestinal chemical barrier②, intestinal immunological barrier③, intestinal physical barrier④, intestinal SCFAs contents⑤, and intestinal microbiota barrier⑥. The red-highlighted indexes indicated their negative relationship with fish intestinal health, whereas the black-highlighted indicated their positive relationship with fish intestinal health. **(D)** On the basis of the Z-score calculated from panel **(C)**, a multidimensional radar map analysis of the effects of five protein treatments on the intestinal health of juvenile largemouth bass was presented. The statistical differences were presented as *P<0.05,**P<0.01, and ***P<0.001..

Pearson’s correlation was used to further study the link between microbiota and physiological indicators (**Figure 8A**). Shannon index, Simpson index, Firmicutes, *Lactococcus*, Anaerobic bacteria, Fusobacteriam, *Plesiomonas*, *Cetobacterium*, and *Aeromonas* were positively correlated with SCFAs (acetic acid, butyric acid, isovaleric acid, isovaleric acid, and propionic acid) and *claudin-4*. In contrast, these two categories of physiological indicators were adversely associated with pathogens, including Proteobacteria, *Pseudomonas*, and Aerobic bacteria. Similarly, Cyanobacteria, Actinobacteria, and *Streptococcus* also had significant positive correlations with ADC, SGR, and *occludin* (*P<0.05*). Inversely, the Verrucomicrobia had adverse correlations with ADC, SGR, and PER (*P<0.05*) although a positive correlation with FCR was observed (*P<0.05*). Surprisingly, the intestinal structure biomarkers were associated with *Weissella (P<0.05)*.

The Mantel test revealed the connections between microbiota/SCFAs, and physiological indicators (**Figure 8B**). Outside of the matrix, *claudin-4* had a significant influence on the abundance of SCFAs and the top ten bacteria at the phylum level (*P<0.05*). In addition, the lipase activity may have a major impact on the top ten microbiota at the genus or phylum level. Within the matrix, the Mantel test revealed the negative association between CAT and villus structure/muscular layer thickness (*P<0.05*), FCR and PER/SGR/occludin (*P<0.05*), lipase and MDA, trypsin and *claudin-1*, LYS and mucin 2 (*P<0.05*). On the other hand, there were positive correlations between villus structure and muscular layer thickness, ADC and *zo-1*/*occludin*/SGR/PER/LPS (*P<0.05*), PER and SGR/*zo-1*/*occludin* (*P<0.05*), goblet cells and LYS/digestive enzymes, CAT and immune globulin/digestive enzymes, GSH-Px and sIgA-like/LYS/digestive enzymes, sIgA-like and digestive enzymes, pepsase and immune globulin/LYS (*P<0.05*), sIgA-like and LYS (*P<0.05*), *claudin-1* and *zo-1* (*P<0.05*).

Next, the Z-score was then computed in **Figure 8C** and presented in **Figure 8D** for a full understanding of the rank of functions for each diet. Briefly, the Z-score value were **microbiota barrier:** CAP/FM (4.87) > CSM/FM (0.89) > TM/FM (-1.29) > FM/FM (-1.36) > ChM/FM (-3.14); **physical barrier:** CAP/FM (4.95) > FM/FM (1.29) > CSM/FM (-0.48) > ChM/FM(-0.68)> TM/FM(-3.3); **immunological barrier:** ChM/FM (2.98)> CAP/FM (1.11) > TM/FM (0.66) > FM/FM (-0.98) > CSM/FM (-3.76); **chemical barrier** ChM/FM (1.68) > CAP/FM (0.62) > TM/FM (-0.27) > CSM/FM (0.91) > FM/FM (-1.11); **antioxidant capacity:** ChM/FM (2.69) > CAP/FM (2.32) > CSM/FM (1.06) > FM/FM (-1.05) > TM/FM (-1.83); **SCFAs contents:** CAP/FM (8.8) > FM/FM (0.12) > ChM/FM(0.11)> CSM/FM (-2.97) > TM/FM (-6.08).

## 4 Discussion

### 4.1 Growth performance, feed utilization and intestinal histological analysis of juvenile largemouth bass under an alternate feeding strategy

It is well known that growth performance is an essential criteria for evaluating fishmeal alternatives. Even though a great deal of research has been conducted on alternate feeding strategy in fish (10, 11, 19), the impacts of new proteins on the growth performance of fish under alternate feeding strategy have not been investigated. In the present research, a substantial decrease in growth performance was detected when comparing diets containing novel proteins to whole fish meal diet; nevertheless, the most surprising discovery was that the growth rate in the second 29 days outperformed the first 29 days. This might be linked to fish-compensatory growth, in which fish that have suffered nutritional deficits instantly undergo a high rate of synthesis upon re-feeding, rapidly raising metabolic levels and enhancing food conversion rates (20). Therefore, the improved growth performance in the second stage of the current study was enhanced, and this was similar to studies done by Zhu et al., 2022, Bi et al., 2021, and Yılmaz et al., 2016 (10, 12, 13), which found the re-feeding of fish oil can also improve the decreased growth performance as well as n-3 ∑PUFA caused by linseed oil, beef tallow-based diets, and canola oil in *A. schlegelii*, *S. maximus*, and *D. labrax* respectively. Moreover, growth performance is possibly related with intestinal bacteria. On the basis of Pearson’s correlation analysis, the number of Cyanobacteria, Acitinobacteria, and *Streptococcus* was positively connected with weight increment; however, the abundance of these bacteria was lower in new protein diets than in whole fishmeal diet. Furthermore, the *occludin* was identified as a biomarker for growth performance by the Mantel test, and the lower *occludin* in diets containing novel proteins compared with the complete fishmeal diet may be responsible for the reduced growth performance. These joint analysis may provide new insight into the decreased growth performance in fish. Although lower growth performance was seen throughout the present investigation, the non-significant changes in intestinal structure and villus height/width demonstrated the growth potential of novel protein diets in the long-term alternate feeding.

### 4.2 The oxidative stress of juvenile largemouth bass under an alternate feeding strategy

Antioxidant enzymes are essential parts of the antioxidant defense system and help to reduce oxidative damage. MDA, an indication of oxidative stress injury in animals, is a lipid peroxidation product (21). In addition, it is well known that the *nrf-2*/*keap-1* pathway protects cells from oxidative stress, and the activation of *nrf2* might up-regulate downstream antioxidant genes that serve as a key cellular defense against the cytotoxic consequences of oxidative stress (22). The present research discovered a decrease in the level of *keap-1*, thus the GSH-Px activity in algae protein *C. vulgaris* diet increased. This was similar to the findings of Mohsen et al. (3), Hassnaa et al. (23), and Li et al. (19) when feeding *Oreochromis niloticus* and *M. salmoides* with a meal containing *C. vulgaris* observing the increased antioxidant capacity. This may be attributed to antioxidant bioactive components, which are pigments and vitamin C contained in this diet (24, 25), thus these factors promoted the colonization of oxidative stress tolerant bacteria ultimately leading to an increase in antioxidant capacity of *C. vulgaris* diet. According to several studies (26, 27), cottonseed protein concentrate, in which free-gossypol content was about 243.94mg/kg is detrimental to the intestinal health in grass carp and Nile tilapia. These fish were believed to tolerate anti-nutritional factors better than carnivorous fish. Our study observed decreased CAT activity in this plant protein diet, and this is largely owing to the poor resistance of carnivorous fish species to free-gossypol content in cottonseed protein concentrate diet (19), which caused intestinal inflammation when its level beyond 13.98mg/kg in a carnivorous fish, *Sillago sihama Forsskál* (28). Surprisingly, *gpx* and *sod* was dramatically reduced in algae diet *C. vulgaris* and cottonseed protein concentrate diets compared to whole fishmeal diet, although their GSH-Px as well as T-SOD enzyme activity was unaffected. In addition, the *keap*/*nrf-2* ratio did not differ between the diets containing novel proteins and the whole fishmeal diet. These results indicated that the antioxidant system may inhibit the expression of these genes through feedback control (29).

### 4.3 The intestinal physical, immunological, and chemical barriers of juvenile largemouth bass under an alternate feeding strategy

The intestinal tight junction genes (*claudin-1*, *zo-1*, *occludin*, and *claudin-4*) and serum IL-1β, TNF-α and LPS can affect intestinal permeability in largemouth bass (17, 30), thus can reflect the intestinal cell-cell integrity. In the current study, the expression of tight-junction genes was suppressed in the cottonseed protein concentrate, algae protein *C. vulgaris*, and insect protein *T. molitor* diets, despite no considerable change in plasma LPS concentration. While the level of *claudin-4* was up-regulated in the bacterial protein *C. autoethanogenum* diet. Additionally, this diet had the lowest LPS level in plasma and intestine compared to other protein diets. However, these results contradicted prior research that claimed the improved physical barrier was detected in yellow worm meal, bacterial protein *C. autoethanogenum*, and algal protein Chlorella diets (17, 19). Possible explanation is that the Gram-negative bacterium, i.e. *Pseudomonas*, which could disrupt the intestinal physical barrier and cause diseases in freshwater fish (31), was most prevalent in whole fishmeal. Therefore, the favorable effects that *T. molitor*, bacterial protein *C. autoethanogenum*, and algal protein *C. vulgaris* diets had were removed during the second fishmeal stage.

Immunity in fish is reliant on the immunological response, which is intimately connected to innate and adaptive immune components such as immunoglobulin (32). In addition, MUC2, LYS, and digestive enzyme can prevent intestine from being invaded by exogenous pathogens (15, 17). These parameters form a critical component of the intestinal immunochemical barrier and play a vital role in maintaining intestinal homeostasis. Our earlier investigation confirmed that a 56-day administration of novel protein diets might increase chemical and immunological intestinal barriers in largemouth bass (17), however the present study did not find any overall changes. Only the content of LYS and sIgA-like rose on the algae protein *C. vulgaris* diet compared with whole fishmeal diet. Similar findings were made by Maliwat et al. (33), Mohsen et al. (3), and Chen et al. (34), who discovered that feeding Chlorella to *Macrobrachium rosenbergii*, *O. niloticus*, and *Oncorhynchus mykiss* increased their immunity. These may be attributed to water-soluble polysaccharides (35), D-Lactic acid (36), and water-soluble alpha-glucans (37) contained in algae protein *C. vulgaris* diet. For other diets containing novel proteins, generally, it seems that the enhanced physiological functions from diets containing novel proteins were overshadowed by the second 29-day fishmeal stage. Also, it is interesting that there was a high correlation between lipase activity and the composition of the microbiota, although there were no significant differences between diets containing novel proteins and whole fishmeal. This is verified in the finding by Yang et al. (38), who also found that supplemental *Aspergillus* lipase can change the microbiota composition in rats. This may be due to the intestine’s enhanced hydrolysis of undigested macronutrients such as proteins and lipids into nutrients useable by the intestinal flora.

### 4.4 The gut microbiota barriers and SCFAs production of juvenile largemouth bass under alternate feeding strategy

The intestinal microbiota may generate toxic compounds that result in barrier abnormality and disease processes, but it could also generate favorable metabolites like SCFAs, which have anti-inflammatory, anti-oxidant, and enteric-epithelial-repair functions that could impact animal wellness and disease progression (39). Thus, the structure and composition of the intestinal microbiota are regarded as crucial for intestinal health, since they improve host physiology and immunity in the intestine and affect body’s immunological response (40). Usually, the dominant bacteria play important role in the health status, and the most abundant bacteria in this research were *Lactococcus*, Firmicutes, *Pseudomonas*, and Protebacteria. Protebacteria, *Pseudomonas*, and *Streptococcus*, which have been recognized as detrimental to aquatic organisms, were shown to be less prevalent in the bacterial protein *C. autoethanogenum* diet. In contrast, a rise in beneficial bacteria, namely *Lactococcus* and Firmicutes, was seen in this diet. Consequently, a significant quantity of acetic acid, butyric acid, and propionic acid were generated. In agreement with the current findings, earlier research has revealed that feeding a diet containing *C. autoethanogenum* increased Firmicutes and decreased Protebacteria in largemouth bass (17, 19, 41). These were also confirmed by the bugbase functional predictions, which indicated that this diet included the greatest concentration of anaerobic bacteria and the lowest concentration of aerobic bacteria. This may be due to the fact that *C. autoethanogenum* contains a significant amount of carbohydrates, lipids, and vitamins that may regulate gut microbiota and enhance intestinal health (6, 42). However, no significant differences in microbiota were identified across other diets containing novel proteins and whole fishmeal diet when using this alternate feeding strategy. A possible explanation for these results may be that the other three protein diets were not as well adapted to the alternate feeding strategy as the bacterial protein *C. autoethanogenum* diet.

SCFAs were recognized to supply energy to intestinal mucosal cells, boost cell metabolism and development, as well as decrease the expansion of pathogens and prevent intestinal dysfunction. The recent research discovered very favorable associations between SCFAs and Shannon/Simpson. These indicators were used to assess the intestinal health (9, 43, 44) in earlier investigations. In addition, our findings were confirmed by a study conducted on mice, which indicated that increasing α-diversity linked to higher levels of short-chain fatty acids (45). Our discovery provides new insight into the elevated intestinal SCFAs in fish, albeit with further study for underlying mechanism. According to the Mantel test, *claudin-4* is important for SCFAs contents, and lower expression was found in yellow meal worm diet, algae protein Chlorella diet, and cottonseed protein concentrate, resulting in lower SCFAs contents in these diets. This finding broadly supported our previous work, which found that tight junctions may affect the production of SCFAs (17).

Pearson’s correlation analysis revealed favorable associations between SCFAs/*claudin-4* and probiotics, suggesting that these bacteria may aid in the production of SCFAs and the maintenance of intestinal barrier integrity. In contrast, the association between Proteobacteria/*Pseudomonas*/Aerobic bacteria and SCFAs yielded contradictory results. These pathogens were previously discovered to cause death or intestinal inflammation in numerous fish, including juvenile hybrid grouper (46) and largemouth bass (17, 30). Nonetheless, significant relationships were also discovered between SCFAs and possible pathogens (*Plesiomonas*/*Aeromonas*). The reason for this is not clear, but it may have something to do with the synergy between microbes, as Ding et al. (47) found the probiotic-treated diet could improve the health status of juvenile *Megalobrama amblycephala* while simultaneously increasing the abundance of both intestinal beneficial bacteria and harmful bacteria.

Despite the fact that physiological and microbial indicators were not significantly altered in the cottonseed protein concentrate diets, yellow worm meal diet, and algae protein *C. vulgaris* diet, compared to the whole fishmeal diet, we sought to assess their function holistically. Therefore, Z-score value of each diet from different physiological biomarkers were computed and summarized. Based on the amount of area occupied by each diet, the order is as follows: bacterial protein *C. autoethanogenum* diet (CAP/FM) > algae protein *C. vulgaris* diet > (ChM/FM) > whole fishmeal diet (FM/FM) > cottonseed protein concentrate diet (CSM/FM) > insect protein *T. molitor* diet (TM/FM). This result is inconsistent with our previous research (17), which indicated that the ranking functions in the algae protein *C. vulgaris* diet (ChM) were the highest, followed by the bacterial protein *C. autoethanogenum* diet (CAP), the insect protein yellow worm meal diet (TM), the fishmeal diet (FM), and the cottonseed protein concentrate diet (CSM). This discrepancy could be attributed to the different feeding strategies and adaptations of juvenile largemouth bass to different novel protein diets. It can thus be suggested that, compared with the whole fishmeal diet, the bacterial protein *C. autoethanogenum* diet and the algae protein *C. vulgaris* diet have better intestinal health under this alternate feeding strategy, which is an effective strategy to feed largemouth bass. However, This alternate feeding technique may not be acceptable for the insect protein *T. molitor* diet or the cottonseed protein concentrate diet.

## 5 Conclusion

**Figure 9:**
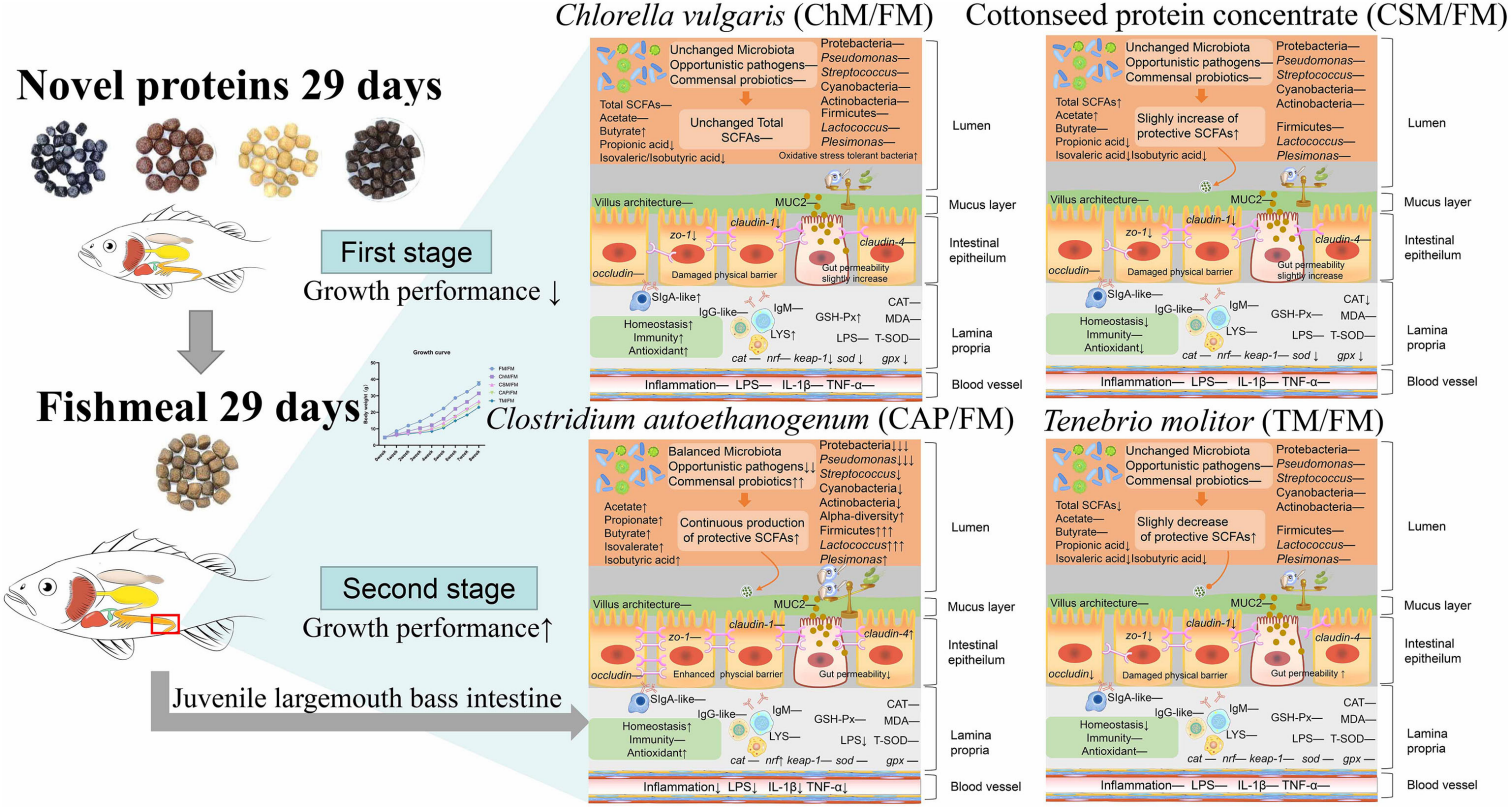
A schematic model showing the mechanism of the effects of novel protein diets on the intestinal health of juvenile largemouth bass under an alternate feeding strategy.

This study set out to evaluate the functions of novel protein diets and a whole fishmeal diet under alternate feeding strategy. From the schematic model of this study (**Figure 9**), the growth performance of all diets containing novel proteins was elevated. In addition, the *C. autoethanogenum* diet and the *C. vulgaris* diet improved intestinal health. However, our comprehensive evaluation of the *T. molitor* diet and the cottonseed protein concentrate diet using the Z-score value revealed that intestinal health performance was inferior to that of the whole fishmeal diet. This work contributes to the existing application of novel protein diets by providing an alternate feeding strategy. Nonetheless, the alternate feeding strategy should be introduced with caution when fed novel proteins, since not all diets performed better than a fishmeal diet when employing this strategy. Future investigation and experimentation into the *C. autoethanogenum* diet as well as the *C. vulgaris* diet, when introduced with alternate feeding, is strongly recommended.

## Data availability statement

The data presented in the study are deposited in the NCBI repository, accession number PRJNA932585.

## Ethics statement

The animal study was reviewed and approved by Chinese order no. 676 of the state Council revised 1 March, 2017. Written informed consent was obtained from the owners for the participation of their animals in this study.

## Author contributions

LL: conceptualization, data curation, formal analysis, investigation, methodology, writing - original draft. YW: sampling, resources, and software. YH: funding. CW: supervision, project administration, visualization writing, editing. All people who made significant contributions to the work presented in the article, including those who offered editing and writing assistance but are not authors, are acknowledged in the paper’s Acknowledgments section and have given their written permission to be included. All authors contributed to the article and approved the submitted version.
