# Supplementary material for: Multi-omics approach to study the dual effects of novel proteins on the intestinal health of juvenile largemouth bass (Micropterus salmoides) under an alternate feeding strategy
Source: Front Immunol. 2023 Mar 1;14:1110696. doi: 10.3389/fimmu.2023.1110696 (PMC10014712; doi:10.3389/fimmu.2023.1110696)
Supplement: Supplementary file 1 [file DataSheet_1.doc]

**Supplementary methods**

**Microbiome study based on sequencing of the 16S rRNA gene**

Considering the biological replicates, Two sample from FM/FM and one sample from CSM/FM were discarded due to its severely deviate from the other samples in the same diet. Total bacterial DNA was yielded from the gut samples using the OMEGA DNA Kit (D5625-01) according to the manufacturer's guidelines (Omega Bio-Tek, Norcross, GA, USA). Using a NanoDrop ND-1000 spectrophotometer (Thermo Fisher Scientific, Waltham, MA, USA) and agarose gel electrophoresis, the amount and quality of isolated DNAs were determined, respectively. The forward primer 341F (5'-CCTAYGGGRBGCASCAG-3') and the reverse primer 806R (5'-GGACTACNNGGGTATCTAAT-3') were used to amplify the 16S rDNA V3-V4 regions. The Quant-iT PicoGreen dsDNA Assay Kit (Invitrogen, Carlsbad, CA, USA) and Vazyme VAHTSTM DNA Clean Beads (Vazyme, Nanjing, China) were used to purify and quantify PCR amplicons. After measuring individual amplicons, they were combined in equal proportions and pair-end 2x250 bp sequencing was carried out at Shanghai Personal Biotechnology Co., Ltd utilizing the Illlumina MiSeq platform and MiSeq Reagent Kit v3 (Shanghai, China). All the bioinformatics analyses were performed by the genescloud tools, based on QIIME2 2019.4 (Bolyen et al., 2019) and R programs (v3.6.1) (https://www.genescloud.cn). The demux plugin was used to demultiplex the raw sequences, and the cutadapt plugin was used to cut the primers (Martin, 2011). The DADA2 plugin was used to perform quality filtering, denoising, merging, and chimera removal on the resulting sequences (Callahan et al., 2016). Each deduplicated sequence created by DADA2's quality control procedure was referred to as an amplicon sequence variation (ASV) with a 100% sequence similarity. The feature-classifier plugin was used to do taxonomic categorization of ASVs using the SILVA Release 132 database and the classify-sklearn naïve Bayes taxonomy classifier (Bokulich et al., 2018).

**Measurement of SCFAs**

Gas chromatography–mass spectrometry (GC–MS) was used to detect fecal SCFAs. In short, 100 mg of sample from each group was weighed and incubated for two minutes at 4°C with 1 mL 0.005 M NaOH solution and 50 µL 2-methyl-butyric acid. Following that, the mixture was mixed for two minutes and centrifuged for twenty minutes (4℃, 13,000 rpm). 500 µL supernatant was transferred to a centrifuge tube and derivatized with 300 µL distilled water, 500 µl isopropanol/pyridine solution (3:2, v/v), and platelet cytotoxic factor solution, followed by 500 µl n-hexane extraction for analysis. Chromatographic separation was performed using an Agilent HP-5 capillary column (30 m*0.25 mm*0.25 m). Samples were injected with a split ratio of ten to one and a volume of 1 µL. The temperatures of the input, the ion source, and the transfer line were 280°C, 230°C, and 250°C, respectively. The initial temperature of the program heating was 60°C, which was retained for five minutes before being increased to 250°C at a rate of 10°C/min. The flow rate of the helium carrier gas was set to 1.0 mL/min. Agilent's MSD ChemStation (E.02.00.493, Agilent Technologies, Inc., USA) was used to manage the data

**Supplementary Table**

**Supplementary Table 1** Experimental feed formulation and proximate composition

| Ingredients | Dietary level (%) | | | |  |
| --- | --- | --- | --- | --- | --- |
|  | FM | CAP | ChM | CSM | TM |
| Fish meal^a^ | 59.14 | 0 | 0 | 0 | 0 |
| *Clostridium autoethanogenum*^b^ | 0 | 49.11 | 0 | 0 | 0 |
| *Chlorella vulgaris*^c^ | 0 | 0 | 74.05 | 0 | 0 |
| *Tenebrio molitor*^d^ | 0 | 0 | 0 | 0 | 64.63 |
| Cottonseed protein concentrate^e^ | 0 | 0 | 0 | 67.05 | 0 |
| Cassava starch | 13 | 13 | 13 | 13 | 13 |
| Wheat gluten | 5 | 5 | 5 | 5 | 5 |
| Fish oil | 3.42 | 5.99 | 2.26 | 5.25 | 4.68 |
| Soybean oil | 3.42 | 5.99 | 2.26 | 5.25 | 4.68 |
| Microcrystalline cellulose | 13.61 | 18.5 | 1.02 | 2.04 | 5.6 |
| Premix^f^ | 1 | 1 | 1 | 1 | 1 |
| Choline chloride | 0.3 | 0.3 | 0.3 | 0.3 | 0.3 |
| Ca(H_2_PO_4_)_2_ | 1 | 1 | 1 | 1 | 1 |
| Y_2_O_3_ | 0.1 | 0.1 | 0.1 | 0.1 | 0.1 |
| BHA | 0.01 | 0.01 | 0.01 | 0.01 | 0.01 |
| Total | 100 | 100 | 100 | 100 | 100 |
| Proximate composition (% dry matter) | | | | |  |
| Moisture | 6.95 | 7.75 | 7.83 | 9.78 | 7.46 |
| Crude protein | 46.06 | 46.68 | 46.37 | 47.07 | 47.93 |
| Crude lipid | 11.80 | 11.48 | 10.07 | 10.71 | 10.43 |
| Ash | 11.24 | 3.84 | 5.21 | 6.60 | 7.09 |
| Gross energy (MJ/kg)^g^ | 20.71 | 21.81 | 21.22 | 20.86 | 21.19 |

^a^Fish meal：TASA super fish meal (Peru)

^b^*Clostridium autoethanogenum*: comes from Hebei Shoulang New Energy Technology Co., Ltd (Hebei, China)

^c^*Chlorella vulgaris*: Institute of Hydrobiology, Chinese Academy of Sciences (Hubei, China)

^d^*Tenebrio molitor*: Guangdong zehecheng Biotechnology Co., Ltd. (Guangdong, China)

^e^Cottonseed protein concentrated: Jinlan plant protein Co., Ltd. (Xinjiang, China)

^f^Vitamin and mineral premix(mg/kg diet): vitamin A, 20; vitamin B_2_, 15; vitamin B_6_, 15; vitamin B1, 10; vitamin B12, 8; vitamin D_3_, 10; vitamin E, 300; vitamin K_3_, 20; inositol 150; niacinamide, 80; calcium pantothenate, 40; biotin, 2; folic acid, 10; FeSO_4_·H_2_O, 300; ZnSO_4_·H_2_O 200mg; NaCl, 100; MnSO_4_·H_2_O, 25; CuSO_4_·5H_2_O, 30; CoCl_2_·6H_2_O(10%Co), 5; Na_2_SeO_3_(10%Se), 5; KIO_3_(2.9%), 3; MgSO_4_·7H_2_O, 1200; zeolite powder 6050.

^g^Gross energy (MJ/kg) = (crude protein×23.6 + crude lipid×39.5 + carbohydrate×17.2)/1000

Supplementary Table 2 Primer sequences for real-time PCR

| Genes | Primer sequence(5'-3') | Tm (℃) | E-values (%) | Sources |
| --- | --- | --- | --- | --- |
| ***β-actin*** | F：ATCGCCGCACTGGTTGTTGAC | 56.2 | 105 | **(Xie et al., 2017)** |
|  | R：CCTGTTGGCTTTGGGGTTC |  |  |  |
| ***occludin*** | F：GATATGGTGGCAGCTACGGT | 57.5 | 91 | **(Zhou et al., 2021)** |
|  | R：TCCTACTGCGGACAGTGTTG |  |  |  |
| ***claudin-1*** | F：CCAGGGAAGGGGAGCAATG | 62.4 | 91 | **(Zhou et al., 2021)** |
|  | R：GCTCTTTGAACCAGTGCGAC |  |  |  |
| ***zo-1*** | F：ATCTCAGCAGGGATTCGACG | 59.9 | 92 | **(Zhou et al., 2021)** |
|  | R：CTTTTGCGGTGGCGTTGG |  |  |  |
| ***claudin-4*** | F：TAATCGCTATGGTGGGAGCC | 61.6 | 107 | **(Zhou et al., 2021)** |
|  | R：GCCCCGATCTCCATCTTCTG |  |  |  |
| ***sod*** | F：CCCCACAACAAGAATCATGC | 58 | 92 | **（zhao et al., 2022）** |
|  | R：TCTCAGCCTTCTCGTGGA |  |  |  |
| ***cat*** | F：GTTCCCGTCCTTCATCCACT | 60.4 | 100 | **（zhao et al., 2022）** |
|  | R：CAGGCTCCAGAAGTCCCACA |  |  |  |
| ***gpx*** | F：CCCTGCAATCAGTTTGGACA | 58.0 | 115 | **（zhao et al., 2022）** |
|  | R：TTGGTTCAAAGCCATTCCCT |  |  |  |
| ***keap-1*** | F：GCACCTAACCGTGGAACTCAA | 60.1 | 93 | **(Zhou et al., 2021)** |
|  | R：CCAGTTTTAGCCAGTCATTGTTCC |  |  |  |
| ***nrf-2*** | F：CCACACGTGACTCTGATTTCTC | 66.6 | 95 | **(Zhou et al., 2021)** |
|  | R：TCCTCCATGACCTTGAAGCAT |  |  |  |
